# Enhancing Self-Care Consultation Skills in Pharmacy Education: Benefits of Virtual Patients and Artificial Intelligence—A Scoping Review

**DOI:** 10.3390/pharmacy14030071

**Published:** 2026-05-11

**Authors:** Radiana Staynova, Daniela Kafalova, Evelina Gavazova, Katerina Slavcheva, Nelina Neycheva, Adelina Boyanova, Desislava Andonova, Hristina Stoynova

**Affiliations:** 1 Department of Organisation and Economics of Pharmacy, Faculty of Pharmacy, Medical University of Plovdiv, 4002 Plovdiv, Bulgaria; 2 Pharmacy Lily, 8800 Sliven, Bulgaria; 3 CLINECA AD, 4002 Plovdiv, Bulgaria

**Keywords:** pharmacy education, self-medication, self-care, communication, virtual patients, artificial intelligence, computer-based simulation, large language models

## Abstract

Virtual patients (VPs) and artificial intelligence (AI) are being implemented in pharmacy education across various countries in order to learn different techniques to improve communication skills, identify drug-related problems, assess the pharmacist’s role in the self-medication process or assess students’ knowledge acquisition. The objective of this study was to assess the benefits of integrating VPs and AI in pharmacy education, particularly their impact on pharmacy students’ knowledge and skills in self-medication counselling. A literature search was conducted across PubMed, Scopus and Web of Science databases. Studies focused on the integration of VPs and AI tools in pharmacy education and their impact on students’ knowledge, counselling and communication skills related to self-medication, were evaluated. Eligible studies were full-text, peer-reviewed research articles published in English. No restrictions were applied regarding publication year. A total of 857 articles were identified through electronic databases and 9 met the inclusion criteria. Six studies were conducted in the USA and one each in Portugal, Sweden and Indonesia. Most studies employed a pre–post-study design. Six studies utilized VP simulations, while the remaining three implemented AI-based tools. Key outcomes covered in analyzed articles included improvements in knowledge score, communication, and consultation skills, along with positive perceptions, including increased student satisfaction and confidence levels. Using VP simulations and AI tools in pharmacy education could positively impact students by enhancing their knowledge as well as their confidence and counselling skills.

## 1. Introduction

The content of pharmacy degree programs is increasingly focused on equipping graduates not only with pharmaceutical knowledge but also with critical thinking and problem-solving skills to manage a variety of medical conditions [1]. Pharmaceutical education worldwide is structured in different ways, with the curricula content determined by healthcare needs across regional, national, and international contexts, the scope of pharmacy practice, career pathways available to graduates, and the existing legal and regulatory frameworks [1,2]. Pharmacists not only dispense medications but can provide a range of primary healthcare services focused on medication safety, disease state management, patient wellness, health screenings, lifestyle modification and medication education [3].

**Figure 1:**
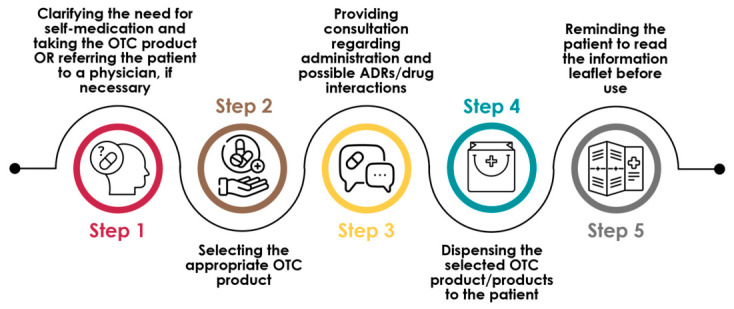
Selection process of non-prescription medicines for self-care.

A particularly critical area for emerging pharmacists is providing counseling to patients seeking non-prescription medicines for self-care (Figure 1) [4]. The World Health Organization (WHO) defines self-care as “the ability of individuals, families and communities to promote health, prevent disease, maintain health and to cope with illness and disability with or without the support of a healthcare provider” [5]. While self-care offers both advantages and challenges, in countries where pharmacists are not authorized to prescribe or participate in the selection of prescription medications, self-medication represents a key opportunity for pharmacists to demonstrate their expertise in pharmacotherapy [6]. A 2011 study by the Pharmaceutical Group of the European Union (PGEU) showed that PGEU members supported the community pharmacists’ core responsibility of assisting patients in management of their self-care [7]. Consequently, pharmacy students need to acquire a comprehensive foundation of drug knowledge to meet advanced pharmacy practice competencies. This educational need can be supported through the use of virtual patient simulations and artificial intelligence-based learning tools.

Virtual patients are increasingly utilized in the education of pharmacy students across multiple countries, supporting a range of learning activities. These include the development of pharmacist–patient communication skills, identification of drug-related problems, management of case-based scenarios involving diverse patient populations, evaluation of the pharmacist’s role in self-medication, and assessment of students’ acquired knowledge [8]. A virtual patient (VP) is defined as an interactive computer simulation of a computer-programmable patient (or avatar) in a real-life clinical scenario for the purposes of medical or pharmacy training [9]. VP is one of the methods of computer-aided learning (CAL) [9]. Studies show that CAL is significantly more successful than traditional teaching methods in improving students’ knowledge, their learning outcomes achievements and satisfaction [10]. The first study reporting the use of VP technology in pharmaceutical education was published in the early 1990s [11]. VP technology has the potential to be an innovative and effective tool in pharmaceutical education [11]. It is argued that using cases involving any of the three types of patients (real, simulated, virtual) is the optimal way to assess students’ critical thinking skills, compared to paper-based case scenarios [12,13]. A growing number of pharmacy institutions worldwide have integrated VP simulations as a pedagogical tool implemented in different courses of the pharmacy curriculum to enhance teaching and learning outcomes [14]. Systematic reviews consistently report the educational benefits of incorporating VPs and computer-based simulations into pharmacy curricula [8,11,14,15].

Artificial intelligence (AI) represents another emerging technology that can be implemented into pharmacy education to strengthen students’ competencies in self-medication consultations. The integration of these new technologies has the ideal potential to improve the performance of students. Additionally, it allows students to have casual conversations while receiving immediate responses [16]. Studies of modern digital pedagogy encourages the use of AI training in professional healthcare education, especially for the development of clinical cognitive skills [17]. There are significant advantages but also some challenges in using AI in pharmacy education [18]. The engagement with the AI tools provides students safe practice and prepares them to work with real patients [19]. The AI methods could help create educational resources that connect theory to practice. This AI use can also facilitate the evaluation process, which will ease the workload for teachers and allow for more regular and impartial evaluations [20]. Further advantages include improving students’ confidence and developing communication abilities that are essential for completing the pharmaceutical education program [20,21]. According to a study, the availability of AI tools and applications has expanded opportunities for pharmacy students, but there are also worries about ethical questions and the dependability and correctness of content produced by AI [22]. Given its growing relevance, AI is poised to become a critical component of pharmaceutical education, making it essential to promote awareness and preparedness among educators and students alike [23].

The benefits of virtual simulations in pharmacy education are widely recognized; however, their specific impact on self-care training and non-prescription counselling represents a significantly under-researched area.

The objective of this study was to assess the benefits of integrating VPs and AI in pharmacy education, particularly their impact on pharmacy students’ knowledge and skills in self-medication counselling.

## 2. Materials and Methods

The current review followed the Preferred Reporting Items for Systematic reviews and Meta-Analyses extension for Scoping Reviews (PRISMA-ScR) guidelines [24]. A completed PRISMA-ScR checklist is provided as Supplementary Material. This scoping review was not prospectively registered in a public registry. Nonetheless, it was conducted in accordance with established methodological frameworks for scoping reviews and reported following PRISMA-ScR guidelines to ensure transparency and reproducibility.

### 2.1. Search Strategy

A comprehensive literature search was conducted using PubMed, Scopus and Web of Science databases from inception to 31 January 2026. The search strategy included the following keywords: (“self-medication” OR “self-care”) AND (“virtual patient” OR “virtual simulation” OR “artificial intelligence” OR “AI”) AND (“pharmacy students” OR “pharmacy education”).

### 2.2. Eligibility Criteria

**Figure 2:**
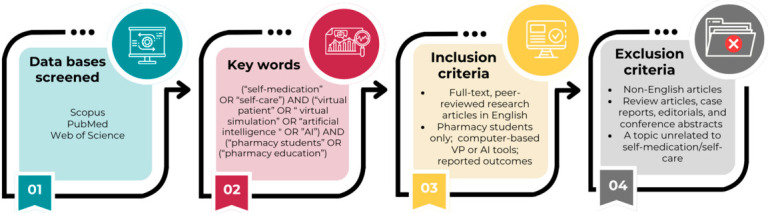
Search process and eligibility criteria.

In this scoping review, only original research articles were considered. The searches were limited to English-language peer-reviewed studies. To be eligible for inclusion, the studies were required to meet the following criteria: (i) involve only pharmacy students; (ii) employ computer-based VP simulation or AI tools within self-medication/self-care education; and (iii) provide detailed outcome measures, such as improvements in knowledge, consultation, confidence, communication skills, or student perceptions and satisfaction (Figure 2). No restrictions were applied regarding publication year.

**Figure 3:**
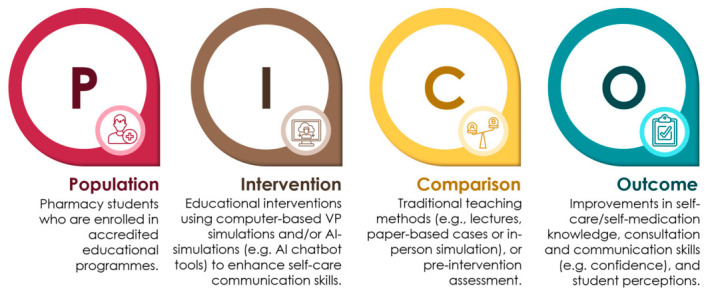
PICO framework for study selection.

Non-English publications and studies that did not address the primary outcomes of this review were excluded. Systematic reviews, meta-analyses, narrative reviews, case reports, editorials, and conference abstracts were also excluded. In addition, studies employing only standardized patients (e.g., human actors) without the use of computer-simulated VPs or AI technologies were not considered. Research involving medical, dental, or nursing students was excluded to maintain a pharmacy education focus. The eligibility criteria were structured according to the PICO framework (Population, Intervention, Comparison, and Outcome), as illustrated in Figure 3.

### 2.3. Study Selection and Data Extraction

The initial reviewing process in the selected databases was conducted by the principal researcher (R.S.). In the second stage, all records were imported into Zotero software v. 6.0.37. for duplicate identification and removal. Then, the titles and abstracts were independently screened by two authors (R.S. and K.S.) to identify potentially relevant studies. The full texts of the included articles were analyzed by seven authors (R.S., K.S., A.B., D.A., E.G., N.N. and H.S.). The full text of studies that were considered potentially relevant were then retrieved and reviewed by the first author (R.S.) to check their eligibility. Disagreements were resolved by discussion with another reviewer (D.K).

A standardized data extraction form was developed, and data were charted using Microsoft Excel^®^. The following data were collected:

(1) Primary author, publication year, country of origin;
(2) Study objective;
(3) Study design;
(4) Sample size, participants and setting;
(5) Technology type (VPs and/or AI);
(6) Self-care topics covered;
(7) Communication and consultation skills assessed;
(8) Students’ assessment and key outcomes.

### 2.4. Critical Appraisal

In line with scoping review methodology and PRISMA-ScR recommendations, critical appraisal of the included studies was not conducted, given the exploratory nature of the review and the heterogeneity of the included study designs.

## 3. Results

**Figure 4:**
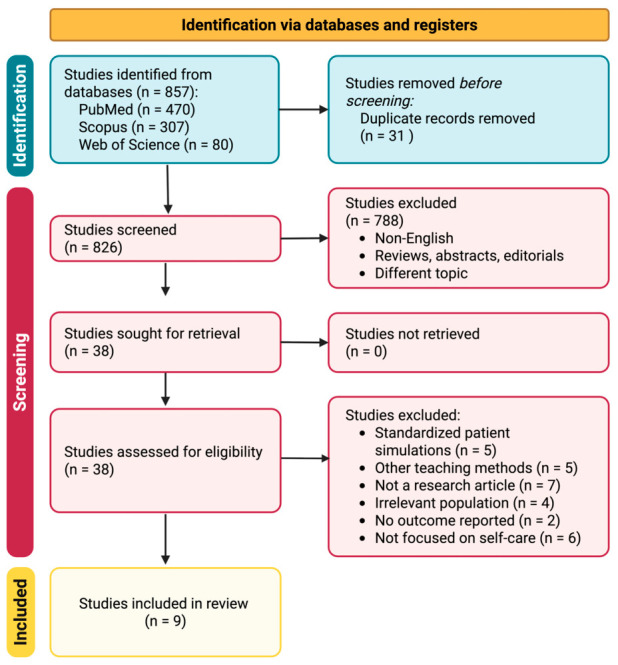
PRISMA-ScR flowchart for study selection.

The initial systematic search across all databases captured 857 papers. Following the removal of duplicates, a total of 826 unique articles were identified. After applying the pre-defined inclusion and exclusion criteria, nine studies were found to meet the eligibility requirements and were included in this scoping review [20,25,26,27,28,29,30,31,32] (Figure 4).

The characteristics of the included studies are summarized in Table 1.

### 3.1. Main Characteristics of Studies Included in the Review

In terms of study design, the identified research was primarily characterized by pre–post evaluations (*n* = 4) [20,27,30,31], followed by quasi-experimental, non-randomized cohort studies (*n* = 2) utilizing historical controls [29,32]. The remaining works consisted of two cross-sectional studies (*n* = 2) [28] and a mixed-methods feasibility study (*n* = 1) [25].

**Figure 5:**
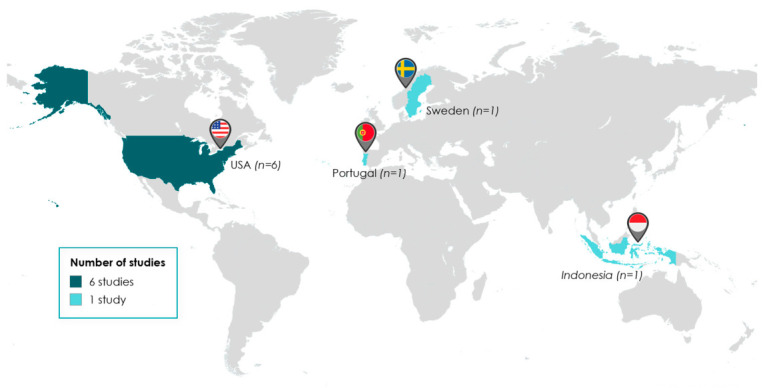
Geographical distribution of studies included in the scoping review (Created with MapChart Version 7.7.1 and Office 365 PowerPoint Version 16.0).

Analysis of the publication data identified the United States as the primary contributor to the field, accounting for six of the included studies [20,27,29,30,31,32]. The remaining research was conducted in Portugal (*n* = 1) [28], Sweden (*n* = 1) [25] and Indonesia (*n* = 1) [26], as illustrated in Figure 5.

Regarding institutional involvement, the majority of the identified research (66.7%, *n* = 6) comprised single-center studies [20,25,26,27,29,32] The remaining proportion involved multiple educational institutions [30,31], with the broadest collaboration observed in the study by Pereira and Cavaco, which included eight distinct pharmacy schools [28].

**Figure 6:**
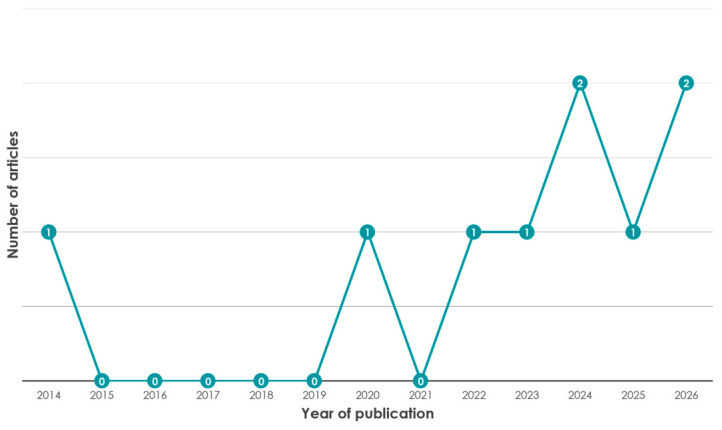
Temporal distribution of studies (2014–2026).

The integration of VPs and AI tools into pharmacy curricula, specifically regarding self-medication counselling and communication skills, was unaddressed in the literature until 2014 (Figure 6). Following a five-year period of research inactivity (2015–2019), the field experienced a concentrated period of growth. Consequently, the vast majority of identified research (*n* = 8) has been published recently, specifically within the period 2020–2026.

### 3.2. Students’ and Courses’ Characteristics

Collectively, the studies enrolled a total of 1743 pharmacy students. Participant cohorts exhibited significant heterogeneity, with individual study sample sizes ranging from a minimum of 9 [25] to a maximum of 717 students [28]. Regarding academic level, most studies focused on students in the early stages of pharmacy education, defined as the first three years of either an undergraduate-entry pharmacy program such as a BPharm or a graduate-entry professional program such as a PharmD *(n* = 7) [20,26,27,29,30,31,32]. One study included participants across a broader range of training, from early-year students to those in the final stages of their pharmacy program (Years 1–5), including internship-level students [28]. In one study, the academic stage of participants was not specified [25]. Given international differences in pharmacy education systems, academic level was interpreted based on relative stage of training rather than strictly by year numbering.

VPs and AI tools were integrated in various pharmacy courses, including Pharmacotherapy and Communication Courses in Portugal [28], The Introductory Pharmacy Practice Experiences (IPPEs), Clinical Skills in Pharmacy Practice, different nonprescription pharmacotherapy courses in the USA [20,27,29,30,31,32], Responding to Symptoms Course in Indonesia [26], and two mandatory self-care courses in Sweden [25].

### 3.3. Clinical Scenarios and Therapeutic Scope

The scenarios implemented in the VP simulations encompassed a range of minor health conditions commonly managed with over-the-counter (OTC) medications. These included the common cold, headache, gastrointestinal disorders (e.g., heartburn, dyspepsia, constipation, diarrhea, nausea, vomiting, and hemorrhoids), allergies, ophthalmic and optic conditions, contraception, back pain, pinworm infection, and smoking cessation.

### 3.4. Simulation Software and AI Tools

Six studies employed VP simulations, whereas the remaining three utilized AI-based tools. Among the studies implementing VPs, two different simulation platforms were identified, including MyDispense (*n* = 5) [27,29,30,31,32] and custom-build virtual interactive simulator named *Simulador de Atendimento*—*SAF* (*n* = 1) [28]. In the three remaining studies included in this scoping review, AI-based tools were applied [20,25,26]. In the study by Khartabil et al., AI-driven virtual patient profiles were developed using Convai.ai to simulate self-care scenarios, with the aim of enhancing students’ communication skills [20]. In addition, Bakhaya et al. employed ChatGPT (GPT-4o) to develop interactive learning models, including simulated patients and automated feedback mechanisms [25]. Brata et al. also used LLMs (Llama 3.1) to developed AI chatbot for Self-Medication Consultation Learning (SMCL-chatbot) [26].

### 3.5. Pharmacy Mnemonics Incorporated in Simulations

Different mnemonics were adopted in the simulation cases across six of the included studies. In the Simulador de Atendimento developed by Pereira and Cavaco, the counseling structure and scenario content followed the WWHAM mnemonic, a framework widely used by community pharmacists in the United Kingdom [28]. This mnemonic has been widely promoted as a decision-support tool for both information gathering and the appropriate supply of non-prescription medicines. The WWHAM framework represents a key component of the self-medication process; however, additional, condition-specific questions may also be necessary to ensure optimal assessment and management of minor ailments [33].

Three other studies employing MyDispense incorporated the QuEST SCHOLAR-MAC mnemonic [27,30,31]. This framework provides a standardized approach for the systematic assessment of patient symptoms, clinical history, and aggravating or alleviating factors. By supporting a comprehensive evaluation of medication use, allergies, and comorbidities, it enables students to determine clinical eligibility for self-care, select appropriate pharmacological interventions, and deliver effective therapeutic counseling [31].

Similarly, the AI-driven case scenarios developed by Khartabil et al. required students to conduct structured patient interviews and apply clinical reasoning to identify potential causes of symptoms and recommend appropriate treatments in accordance with the SCHOLAR-MAC framework [20].

The large language model-based feedback system developed by Bakhaya et al. focused on key communication skills relevant to self-care consultations. This system was based on a consultation framework commonly used in pharmacy education and practice in Sweden, known as “The communication ladder”. This framework comprises four sequential phases designed to systematically identify and address patients’ self-care needs [25].

**Figure 7:**
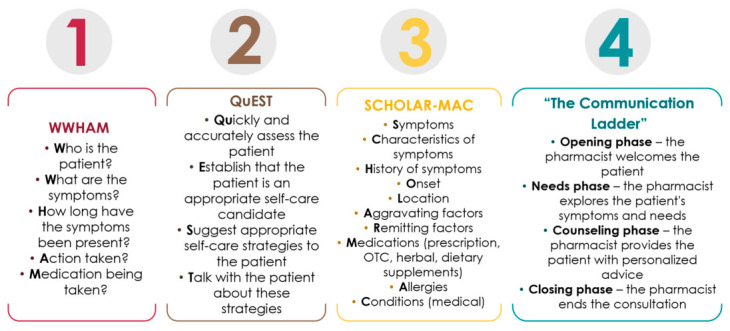
Structure of mnemonics and frameworks incorporated in simulation scenarios.

The structure of all mnemonics and frameworks integrated into the simulation scenarios is presented in Figure 7.

### 3.6. Educational Benefits

#### 3.6.1. Impact of Simulation on Students’ Knowledge

Pharmacy students’ knowledge was evaluated across 3 studies [27,30,31], all of which used pre–post surveys to measure changes before and after the activity with VPs. Rude et al. demonstrated a significant improvement in total knowledge scores among 142 students from two institutions who completed both the pre- and post-surveys following engagement with VPs [31]. Similarly, Riskin et al. reported a statistically significant increase in knowledge (*p* < 0.001) among a cohort of 198 third-year pharmacy students from three institutions after participation in a virtual self-care simulation [30]. In a crossover study comparing in-person simulation with asynchronous, at-home virtual simulation, Donohoe et al. found that total knowledge scores improved following both modalities [27].

#### 3.6.2. Impact of Simulation on Counselling Skills and Self-Confidence

Another key outcome assessed in the analyzed studies was the improvement of counselling skills and self-confidence. Riskin et al. reported a significant increase in student confidence following a virtual self-care simulation, both for the overall cohort and within individual institutions. The average self-confidence ratings were in the mid-80s on a 0–100 scale for each assessed item [30]. Similarly, Rude et al. observed significant improvements across all five measured confidence statements after students completed an OTC simulation using virtual patients [31].

Pereira and Cavaco found that pharmacy students achieved acceptable counseling performance after training with the VP simulator SAF, with a mean score of 8.03 (SD = 1.25), exceeding the threshold of 8 out of 10 [28]. In contrast, Mazan et al. reported that the use of MyDispense did not significantly enhance students’ communication skills; however, it did lead to a statistically significant improvement in their ability to recommend appropriate OTC products (*p* = 0.001), suggesting that virtual simulation may be particularly effective for developing product selection competencies [29].

Tai et al. further demonstrated that students who completed virtual simulation cases reported significantly more patient care interactions during IPPEs compared to a control group, although no differences were observed in self-reported confidence [32]. Additionally, Khartabil et al. identified notable increases in student confidence following the use of AI-based tools, alongside a greater willingness to engage with such technologies in clinical learning environments [20]. Most participants also reported that the AI tool was easy to use and contributed to improved confidence in communication skills [20].

#### 3.6.3. Students’ Perceptions and Satisfaction

Six studies reported pharmacy students’ perception of VPs and AI tools [20,25,26,27,30,31].

In the study of Khartabil et al., students reported positive experiences and highlighted the ease of use of the technology for practicing communication skills. Many participants noted that the AI tool was intuitive and easy to navigate, requiring minimal time to achieve proficiency. This usability enabled students to concentrate on developing their communication skills rather than managing complex software interfaces. Perceived benefits included improved communication abilities, increased confidence, and valuable practice opportunities that simulate real-world pharmacy scenarios [20].

Similarly, in a study evaluating MyDispense, students expressed positive perceptions of the activity, indicating that the OTC simulation encouraged them to engage with the material in novel ways and that they would recommend the activity to other students. Most participants perceived improvements in their OTC knowledge and skills, and emphasized that the platform facilitated the meaningful application of newly acquired knowledge [30].

Consistent with these findings, Rude et al. reported overall positive student perceptions of activities involving VPs, with high levels of satisfaction and perceived benefits for both knowledge acquisition and skill development [31].

In contrast, Donohoe et al. reported that students expressed a preference for in-person learning sessions over virtual, asynchronous self-care simulations delivered via MyDispense. Nevertheless, the authors concluded that in resource-constrained settings where facilitators and/or budget are limited, asynchronous activities may represent a practical alternative [27].

To evaluate Indonesian pharmacy students’ intentions to use an interactive AI chatbot for Self-Medication Consultation Learning (SMCL-chatbot), Brata et al. administered a questionnaire adapted from the Unified Theory of Acceptance and Use of Technology (UTAUT2) [26]. The findings indicated that a substantial proportion of students (90%) reported a positive intention to use the SMCL-chatbot, while more than 80% expressed favorable perceptions across key UTAUT2 constructs, including performance expectancy, effort expectancy, facilitating conditions, and hedonic motivation. The multivariate analysis revealed that performance expectancy (OR: 16.5, 95% CI: 1.42–192.42, *p* = 0.025) and hedonistic motivation (OR: 19.4, 95% CI: 2.60–144.63, *p* = 0.004) were significant predictors of students’ intention to use the SMCL chatbot. However, this preliminary study did not assess changes in students’ competence following use of the SMCL-chatbot. Accordingly, the authors emphasized the need for further research to determine whether the AI-based tool can effectively enhance students’ competence in conducting self-medication consultations [26].

Bakhaya et al. further explored students’ perceptions and experiences with LLM-based chatbots [25]. Students found the simulated patients easy to use, which facilitated the initiation and maintenance of conversations. Interactions were described as natural and comparable to everyday messaging platforms. Participants regarded both the simulated patients and the automated feedback system as valuable complements to existing training approaches. Moreover, students perceived chatbot-simulated patients as beneficial for preparing for future professional practice. Most participants found the interactions engaging and interesting, and considered the simulated patient cases to be realistic, underscoring the potential of these tools to support authentic learning experiences [25].

## 4. Discussion

This scoping review provides a summary of the available literature on the use of VPs and AI in pharmacy education, with a particular focus on self-care counselling. The findings suggest that these digital approaches can support the development of students’ knowledge, confidence, and consultation competencies. However, the relatively limited number of identified studies indicates that this area remains insufficiently explored and continues to evolve. Notably, the application of VPs, including AI-based approaches, in relation to communication skills and consultation competencies in self-care contexts has not been extensively addressed in the literature. Only three studies included in this review report the use of AI-driven VPs in pharmacy education specifically related to self-care [20,25,26]. Overall, the findings of this scoping review suggest that VPs and AI-based tools have a positive impact on pharmacy students’ learning outcomes, particularly in terms of knowledge acquisition, confidence, and communication skills in self-care consultations. It should also be noted that the predominance of studies conducted in the United States may limit the generalisability of these findings across different educational and regulatory contexts.

Most of the included studies used pre- and post-intervention assessments and self-reported measures, capturing students’ perceptions rather than objectively measured competencies. Consequently, the reported improvements largely reflect perceived gains rather than demonstrated performance in real pharmacy settings. From an educational perspective, these outcomes correspond primarily to the lower levels of Miller’s pyramid (“knows” and “knows how”), with limited evidence of higher-level competencies in practice. While both VP simulations and AI-based tools show educational potential, VP platforms appear to be more frequently used in the included studies, whereas AI-based tools represent a more recent development. They may offer additional flexibility and opportunities for interactive, real-time communication training, although evidence remains limited. In this context, the findings suggest that these technologies are best positioned as complementary approaches rather than replacements for traditional teaching. Their integration into pharmacy education may enhance learning by providing additional opportunities for practice and feedback in simulated environments, thereby supporting the development of consultation skills.

These considerations should be interpreted in the context of the growing global emphasis on self-care. Within this framework, pharmacists play a key role as one of the most accessible healthcare providers who support patients in the safe and appropriate use of non-prescription medicines. Accordingly, pharmacy education must ensure that students develop not only strong pharmacotherapeutic knowledge but also effective communication and counselling skills to promote safe self-medication practices.

Pharmacists contribute to self-care by assessing patients’ suitability for self-management, selecting appropriate non-pharmacological and pharmacological interventions, and developing individualized care plans [34]. In this context, effective patient counselling is essential to ensure the safe and appropriate use of OTC medicines, which, when used incorrectly, may lead to risks such as misuse and inappropriate dosing [35]. Furthermore, the frequent inclusion of OTC products in medication safety reports highlights the critical role of pharmacist-led counselling in minimizing risks and preventing inappropriate therapy [36]. Studies described in this scoping review suggest that VP simulations and AI-based tools may support the development of counselling skills, clinical decision-making, as well as appropriate OTC selection and recommendations in self-care scenarios. Across the included studies, MyDispense was identified as the most frequently implemented virtual patient simulation platform, suggesting its established role in contemporary pharmacy education [27,29,30,31,32]. Developed at Monash University, MyDispense is an open-source, avatar-based virtual dispensing simulator designed to replicate community pharmacy practice within a safe educational environment [37]. It enables students to develop critical thinking and clinical decision-making skills through activities such as patient interviewing, prescription assessment, and the provision of patient counselling [38]. The platform allows students to perform key dispensing tasks, including patient information verification, prescription validation, and product selection, while applying their knowledge of prescription and OTC medicines in self-care scenarios [39]. Its use has been associated with improvements in academic performance, professional competency, and the ability to identify prescription errors and apply legal requirements in practice [39]. Furthermore, students consistently reported that the platform was user-friendly and offered a more realistic learning experience compared to traditional paper-based case studies, with higher levels of engagement, perceived authenticity, and acquisition of new knowledge [40]. Virtual simulation through MyDispense has also been linked to increased confidence in clinical skills, primarily due to opportunities for repeated practice in a risk-free environment. This setting allows learners to receive feedback and learn from errors without compromising patient safety. Positive perceptions have been reported by both students and practicing pharmacists, who noted enhancements in information gathering, prescription review, OTC recommendations, and patient counselling skills [41,42]. Additionally, students highlighted the critical role of MyDispense during the COVID-19 pandemic, as it supported the continuity of experiential learning when access to in-person training was limited [42]. However, it should be noted that most of the reported outcomes are based on self-reported measures, which may not fully reflect actual clinical competence or performance in real-world settings.

The integration of AI into healthcare education presents considerable pedagogical potential. Current evidence indicates that students perceive AI as a valuable tool for enhancing learning, developing research skills, improving clinical reasoning, and preparing for real-world clinical practice [43]. Students also reported that AI supports academic tasks, research activities, and clinical training by improving learning efficiency and facilitating the understanding of complex topics [43]. Beyond education, AI was generally perceived as a collaborative tool rather than a competitor, with potential to improve patient care, pharmacy services, and the overall efficiency of healthcare systems [44].

In the context of pharmacy curricula, teaching self-care requires various pedagogical strategies. Simulation-based learning has been established as a safe, effective, and well-received educational methodology within health sciences [31]. The implementation of GenAI within these simulated frameworks provides a transformative pathway for augmenting student engagement and fostering active learning. Considering that pharmacy skills training aims to provide students with opportunities to practice in controlled, simulated environments, the integration of AI represents a logical advancement, enabling engagement with emerging technologies in low-risk settings and supporting the development of competencies applicable to real clinical practice [45]. These findings are consistent with previous research, which indicates that despite the growing interest in AI, the proportion of students receiving formal education in this field remains limited, underscoring the need for its broader integration into healthcare curricula [46]. Despite the demonstrated benefits of AI and simulation-based learning, several barriers to their implementation have been identified in the literature. These include resistance to change, financial and time constraints, software usability issues, and challenges related to meeting accreditation standards, as well as limited faculty experience, curriculum constraints, and difficulties in maintaining student motivation and engagement [47].

Future perspectives emphasize the importance of integrating virtual simulation technologies into pharmacy curricula to enhance students’ preparedness for real-world practice. These tools support the development of essential competencies by enabling students to understand the complexity of the dispensing process, including legal, clinical, and counselling aspects. Moreover, repeated practice in a low-risk environment may improve efficiency, reduce errors, and increase students’ confidence when transitioning to real clinical settings [48].

### Strength and Limitations

The present scoping review has several strengths that should be acknowledged. To the best of our knowledge, this study represents the first comprehensive synthesis of evidence concerning the utility of VPs and AI-based tools in enhancing self-care consultation skills within pharmacy education. While self-care counseling is well-established component in pharmacy curricula, the use of VPs and AI to support this training remains underexplored; this review thus provides a critical and up-to-date analysis of these specific educational frameworks. Furthermore, the inclusion of studies from different countries and educational settings offers insights across different educational contexts. The search period was broad enough, without limitations on the date of published articles, and three major databases were searched to find relevant records.

However, several limitations should be considered. Firstly, the relatively small number of included studies, particularly those involving AI-based approaches, restricts the ability to draw definitive conclusions. Secondly, heterogeneity in study designs, sample sizes, and outcome measures complicates direct comparisons. Additionally, the inclusion of only English-language publications may introduce selection bias, while the predominance of studies conducted in the USA may limit generalizability. Furthermore, the current scoping review includes only peer-review articles; non-refereed articles and grey literature were excluded, which may have led to the omission of relevant and potentially important findings.

## 5. Conclusions

This scoping review identified key outcomes across the analyzed studies, namely improvements in knowledge, communication, and consultation skills, along with positive perceptions such as increased student satisfaction and self-confidence. The integration of VP simulations and AI-based tools into pharmacy curricula, particularly in self-care education, appears to provide meaningful educational benefits by enhancing both theoretical knowledge and practical counselling skills. However, further research is needed to confirm these effects and to determine whether they translate into improved performance in real-world pharmacy practice.

## Figures and Tables

**Table 1:** Summary of included studies.

Authors, Year, Country	Study Objective	Study Design	Sample Size, Participants and Setting	Technology (VPs/AI)	Self-Care Topics Covered	Communication and Consultation Skills Assessed	Students’ Assessment and Key Outcomes
Pereira and Cavaco (2014), Portugal [28]	To explore the minor ailment counseling skills of Portuguese pharmacy undergraduates through an experimental virtual patient methodology	Descriptive, prospective cross-sectional study	Pharmacy undergraduates (Years 1–5) and internship students (*n* = 717) from eight Portuguese pharmacy schools	Custom-build Virtual Interactive Simulator—*Simulador de Atendimento* (*SAF*)	Common cold	WWHAM mnemonic was used	Older students and those with prior pharmacy experience demonstrated significantly better counseling performance, while simulator acceptance was consistent across cohorts.
Tai et al. (2020), USA [32]	To evaluate the impact of MyDispense virtual simulation in a first-year self-care therapeutics course on subsequent IPPE self-care encounters	Quasi-experimental non-randomized cohort study (intervention vs. historical control)	First-year pharmacy students, including intervention group (*n* = 22) and control group (*n* = 26) from a single U.S. institution—University of Michigan College of Pharmacy	MyDispense online pharmacy simulator using VPs	Cold, cough, fever, contraception, heartburn, allergies, constipation, diarrhea, nausea/vomiting, acne, musculoskeletal pain, ophthalmic health, oral health.	Skills in patients interviewing and providing appropriate recommendations—either for self-care/OTC products or referral to a healthcare provider—were assessed.	Students in the intervention group reported significantly more patient care interactions during IPPEs than those in the control group. However, there were no differences in self-reported confidence.
Mazan et al. (2022), USA [29]	To evaluate the effectiveness of virtual simulation on pharmacy students’ ability to assess and formulate a plan for patients seeking self-care.	Quasi-experimental non-randomized cohort study (intervention vs. historical control)	Third-year pharmacy students including intervention group (*n* = 135) and control group (*n* = 178) from a single U.S. institution—Midwestern University College of Pharmacy	MyDispense online pharmacy simulator using VPs	Back pain, cold, cough, headache and seasonal allergies	OTC recommendations were provided, including obtaining necessarily information to assess self-medication eligibility, selecting an appropriate OTC product, and delivering patient education. Students selected from predefined questions to gather patient information and typed their counseling points for the virtual patient.	The intervention group was more likely to choose an appropriate product. However, no significant difference was observed in overall scores for providing self-care recommendations between two groups.
Rude et al. (2023), USA [31]	To assess the impact of the OTC simulation on students’ knowledge and confidence of OTC medications and overall perceptions of the activity	Pre–post study	First-year pharmacy students (*n* = 142) from two U.S. institutions—North Dakota State University (NDSU) and Virginia Commonwealth University (VCU)	MyDispense online pharmacy simulator using VPs	Common medical conditions in the areas of ophthalmology, upper gastrointestinal tract (heartburn), pain, allergies, and cough/cold.	QuEST SCHOLAR-MAC framework was used.	Virtual pharmacy simulation resulted in increased student knowledge and confidence in providing OTC recommendations. Student perceptions were overall very positive.
Riskin et al. (2024), USA [30]	To evaluate whether a virtual, self-care activity improved knowledge and confidence in third-year pharmacy students	Multi-institutional pre–post study	Third-year pharmacy students (*n* = 198) from three accredited Colleges of Pharmacy—Nova Southeastern University, Virginia Commonwealth University and North Dakota State University	MyDispense online pharmacy simulator using VPs	Constipation, pain, cough and cold, *tinea pedis*, and allergic rhinitis.	QuEST SCHOLAR-MAC framework was used. Self-reported communication confidence and preparedness was assessed.	Pre–post assessments showed significant improvements in knowledge (*p* < 0.001) for the entire cohort and improved confidence across all institutions, with students reporting positive perceptions of the virtual self-care simulation.
Donohoe et al., (2024), USA [27]	To compare live laboratory and asynchronous virtual sessions, using a crossover design, in improving knowledge and confidence in self-care review topics.	Crossover pre–post study	Third-year pharmacy students (*n* = 67) from a single institution in U.S.—Virginia Commonwealth University School of Pharmacy	MyDispense online pharmacy simulator using VPs	Cough and cold, pain, allergies constipation/diarrhea, hemorrhoids, heartburn, lice, dermatology	QuEST SCHOLAR-MAC framework was used. Assesses patient suitability for self-care and provided appropriate OTC and non-pharmacologic treatment recommendations.	Post-assessment knowledge and confidence significantly increased after both the live, active-learning session and the asynchronous simulation. The majority of students (85%) indicated that they preferred the in-person activity.
Khartabil et al. (2025), USA [20]	To assess pharmacy students’ perceptions and confidence of using AI-based communication tools during virtual patient encounters, and to evaluate their communication skill development.	Pre–post study	First-year pharmacy students (*n* = 48): pre-survey (*n* = 48) post-survey (*n* = 35) Single institution from U.S.	AI-driven virtual patient profiles developed using Convai.ai to simulate self-care scenarios, aiming to enhance students’ communication skills.	Pain, women’s health, oral, ophthalmic and ear conditions	SCHOLAR-MAC format was used. Patient interview and clinical reasoning were applied.	The study concludes that AI communication tools offer valuable opportunities for enhancing pharmacy students’ communication skills.
Bakhaya et al., (2026), Sweden [25]	To examine pharmacy students’ and faculty’s perceptions and the perceived feasibility of employing LLM-based chatbots to support training in written, real-time communication for self-care consultations.	Mixed-methods feasibility study	Pharmacy students (*n* = 9) and Faculty members involved in self-care courses *(n* = 5) from a single institution (Uppsala University). Year of education was not specified.	ChatGPT-based models (GPT-4o)	Cold and cough, pinworms, smoking cessation	“The communication Ladder” framework was used in the LLM-based automated feedback system. Each step from the framework highlights typical pharmacist–patient interactions and communication goals within that phase.	Participants highlighted the authenticity of the simulated interactions, particularly the emotional realism. The AI-generated feedback was regarded as structured, detailed, and fair, and was especially appreciated for its focus on communication skills.
Brata et al. (2026), Indonesia [26]	To describe pharmacy students’ intentions to use an interactive AI chatbot system for self-medication consultation learning	Cross-sectional study	Third-year pharmacy students (*n* = 201) from a single institution (Pharmacy school at the University of Surabaya)	AI chatbot for Self-Medication Consultation Learning (SMCL-chatbot) using Llama 3.1	Chronic cough due to asthma worsening, acute diarrhea in children without alarm symptoms, dyspepsia due to the use of NSAIDs, dyspepsia with alarm symptoms, mild nausea and vomiting in pregnancy, and viral conjunctivitis.	Improvements in students’ competence after using SMCL-chatbot were not assessed.	Students showed positive intentions to use the SMCL-chatbot, indicating their readiness to adopt the technology for learning self-medication consultations.

Abbreviations: IPPEs, Introductory Pharmacy Practice Experiences; LLM, Large Language Model; OTC, Over-the-counter; SMCL, Self-Medication Consultation Learning; WWHAM, W—Who is the patient? W—What are the symptoms? H—How long have the symptoms been present? A—Action taken? M—medication being taken?

## Data Availability

No new data were created or analyzed in this study.

## Supplementary Materials

The following supporting information can be downloaded at: https://www.mdpi.com/article/10.3390/pharmacy14030071/s1, Table S1: Preferred Reporting Items for Systematic reviews and Meta-Analyses extension for Scoping Reviews (PRISMA-ScR) Checklist. File S1: Full Electronic Search Strategy.

## Abbreviations

The following abbreviations are used in this manuscript:

AI	Artificial Intelligence
CAL	Computer-aided Learning
LLM	Large language model
PGEU	Pharmaceutical Group of the European Union
IPPEs	Introductory Pharmacy Practice Experiences
OTC	Over-the-counter
SMCL	Self-Medication Consultation Learning
UTAUT2	Unified Theory of Acceptance and Use of Technology
VP	Virtual Patient
WHO	World Health Organization

## References

[1] Blouin R.A., Joyner P.U., Pollack G.M. (2008). Preparing for a Renaissance in Pharmacy Education: The Need, Opportunity, and Capacity for Change. Am. J. Pharm. Educ..

[2] Katoue M.G., Schwinghammer T.L. (2018). Competency-Based Pharmacy Education: An Educational Paradigm for the Pharmacy Profession to Meet Society’s Healthcare Needs. Pharmacy Education in the Twenty First Century and Beyond.

[3] Ried L.D., Posey L.M. (2006). The Changing Face of Pharmacy. J. Am. Pharm. Assoc..

[4] Gilson A.M., Stone J.A., Reddy A., Chui M.A. (2019). Exploring How Pharmacists Engage with Patients about Over-the-Counter Medications. J. Am. Pharm. Assoc..

[5] Narasimhan M., Allotey P., Hardon A. (2019). Self Care Interventions to Advance Health and Wellbeing: A Conceptual Framework to Inform Normative Guidance. BMJ.

[6] Bell J., Dziekan G., Pollack C., Mahachai V. (2016). Self-Care in the Twenty First Century: A Vital Role for the Pharmacist. Adv. Ther..

[7] PGEU (2012). PGEU Survey on Pharmacy Education in Relation to Non-Prescription Medicines/Self-Care.

[8] Beshir S.A., Mohamed A.P., Soorya A., Sir Loon Goh S., Moussa El-Labadd E., Hussain N., Said A.S.A. (2022). Virtual Patient Simulation in Pharmacy Education: A Systematic Review. Pharm. Educ..

[9] Klemenc-Ketis Z., Cagran B., Dinevski D. (2018). Evaluating the Difference between Virtual and Paper-Based Clinical Cases in Family Medicine Undergraduate Education. Adv. Med..

[10] Schifferdecker K.E., Berman N.B., Fall L.H., Fischer M.R. (2012). Adoption of Computer-Assisted Learning in Medical Education: The Educators’ Perspective: Adoption of Computer-Assisted Learning in Medical Education. Med. Educ..

[11] Jabbur-Lopes M.O., Mesquita A.R., Silva L.M.A., De Almeida Neto A., Lyra D.P. (2012). Virtual Patients in Pharmacy Education. Am. J. Pharm. Educ..

[12] Barnett S.G., Gallimore C.E., Pitterle M., Morrill J. (2016). Impact of a Paper vs. Virtual Simulated Patient Case on Student-Perceived Confidence and Engagement. Am. J. Pharm. Educ..

[13] Bernaitis N., Baumann-Birkbeck L., Alcorn S., Powell M., Arora D., Anoopkumar-Dukie S. (2018). Simulated Patient Cases Using DecisionSim^TM^ Improves Student Performance and Satisfaction in Pharmacotherapeutics Education. Curr. Pharm. Teach. Learn..

[14] Korayem G.B., Alshaya O.A., Kurdi S.M., Alnajjar L.I., Badr A.F., Alfahed A., Cluntun A. (2022). Simulation-Based Education Implementation in Pharmacy Curriculum: A Review of the Current Status. Adv. Med. Educ. Pract..

[15] Phanudulkitti C., Puengrung S., Meepong R., Vanderboll K., Farris K.B., Vordenberg S.E. (2023). A Systematic Review on the Use of Virtual Patient and Computer-Based Simulation for Experiential Pharmacy Education. Explor. Res. Clin. Soc. Pharm..

[16] Pit S.W., Hamiduzzaman M., Schneider C.R., Barraclough F. (2025). Evaluation Framework for Conversational AI Agents in Pharmacy Education: A Scoping Review of Key Characteristics and Outcome Measures. Res. Soc. Adm. Pharm..

[17] Mir M.M., Mir G.M., Raina N.T., Mir S.M., Mir S.M., Miskeen E., Alharthi M.H., Alamri M.M.S. (2023). Application of Artificial Intelligence in Medical Education: Current Scenario and Future Perspectives. J. Adv. Med. Educ. Prof..

[18] Smetana K.S., Postema S., Smetana M.E. (2025). Exploring Pharmacists’ Perceptions of Text-Based Artificial Intelligence in Resident and Student Education. Hosp. Pharm..

[19] Sallam M., Salim N., Barakat M., Al-Tammemi A. (2023). ChatGPT Applications in Medical, Dental, Pharmacy, and Public Health Education: A Descriptive Study Highlighting the Advantages and Limitations. Narra J..

[20] Khartabil N., Laleh K., Coyne L. (2025). Exploring Pharmacy Students’ Perceptions and Confidence in Using AI Communication Tools. Curr. Pharm. Teach. Learn..

[21] Owoseni A., Kolade O., Egbetokun A. (2024). Generative AI and Its Implications for Higher Education Students and Educators. Generative AI in Higher Education.

[22] Abdel Aziz M.H., Rowe C., Southwood R., Nogid A., Berman S., Gustafson K. (2024). A Scoping Review of Artificial Intelligence within Pharmacy Education. Am. J. Pharm. Educ..

[23] Al-Ghazali M.A. (2025). Evaluation of Awareness, Perception and Opinions Toward Artificial Intelligence Among Pharmacy Students. Hosp. Pharm..

[24] Tricco A.C., Lillie E., Zarin W., O’Brien K.K., Colquhoun H., Levac D., Moher D., Peters M.D.J., Horsley T., Weeks L. (2018). PRISMA Extension for Scoping Reviews (PRISMA-ScR): Checklist and Explanation. Ann. Intern. Med..

[25] Bakhaya S., Lehnbom E.C., De Carvalho Filho M.A., Ma K.Y., Svensberg K. (2026). Development and Evaluation of AI Chatbot Tool for Written Communication Training in Self-Care: Experiences of Pharmacy Students and Faculty. Curr. Pharm. Teach. Learn..

[26] Brata C., Wibowo Y.I., Sari G.A.P.L.P., Yana I. (2026). Intention to Use an Interactive Artificial Intelligence (AI) Chatbot for Learning Self-Medication Consultation Among Pharmacy Students. Pharm. Educ..

[27] Donohoe K.L., Eukel H., Riskin J.W., Ahmed-Sarwar N., Ohman T., Sutton E.M., Powers K., Caldas L.M. (2024). A Comparison of In-Person vs. Asynchronous Learning with Self-Care Patient Cases. Curr. Pharm. Teach. Learn..

[28] Pereira D.V., Cavaco A.M. (2014). Exploring Computer Simulation to Assess Counseling Skills Amongst Pharmacy Undergraduates. Indian J. Pharm. Educ. Res..

[29] Mazan J., Komperda K., D’Souza J. (2022). Effects of Virtual Simulation on Student Pharmacists’ Ability to Assess Self-Care Patient Cases. Curr. Pharm. Teach. Learn..

[30] Riskin J.W., Donohoe K.L., Ahmed-Sarwar N., Eukel H., Ohman T., Powers K., Sutton Burke E.M., Caldas L.M. (2024). Virtual Self-Care Simulations for Third-Year Pharmacy Skills Laboratory Courses in Three Institutions. Curr. Pharm. Teach. Learn..

[31] Rude T.A., Eukel H.N., Ahmed-Sarwar N., Burke E.S., Anderson A.N., Riskin J., Caldas L.M. (2023). An Introductory Over-the-Counter Simulation for First-Year Pharmacy Students Using a Virtual Pharmacy. Am. J. Pharm. Educ..

[32] Tai M.-H., Rida N., Klein K.C., Diez H., Wells T., Kippes K., Walker P.C., Vordenberg S.E. (2020). Impact of Virtual Simulation in Self-Care Therapeutics Course on Introductory Pharmacy Practice Experience Self-Care Encounters. Curr. Pharm. Teach. Learn..

[33] Lelie-van der Zande R., Koster E.S., Teichert M., Bouvy M.L. (2021). Allergic Rhinitis Self-Care Advice in Community Pharmacies: A Simulated Patient Study. Explor. Res. Clin. Soc. Pharm..

[34] Rutter P. (2015). Role of Community Pharmacists in Patients’ Self-Care and Self-Medication. Integr. Pharm. Res. Pract..

[35] Straw A., Mills J., Winters R., Van De Roovaart H., Chen A.M.H. (2023). Community Pharmacies and the Empowerment of Self-Care in the United States. Explor. Res. Clin. Soc. Pharm..

[36] Bertsche T., Alexa J.M., Eickhoff C., Schulz M. (2023). Self-Care and Self-Medication as Central Components of Healthcare in Germany—On the Way to Evidence-Based Pharmacy. Explor. Res. Clin. Soc. Pharm..

[37] Mak V., Fitzgerald J., Holle L., Vordenberg S.E., Kebodeaux C. (2021). Meeting Pharmacy Educational Outcomes through Effective Use of the Virtual Simulation MyDispense. Curr. Pharm. Teach. Learn..

[38] Ambroziak K., Ibrahim N., Marshall V.D., Kelling S.E. (2018). Virtual Simulation to Personalize Student Learning in a Required Pharmacy Course. Curr. Pharm. Teach. Learn..

[39] Khera H.K., Mannix E., Moussa R., Mak V. (2023). MyDispense Simulation in Pharmacy Education: A Scoping Review. J. Pharm. Policy Pract..

[40] Johnson A.E., Barrack J., Fitzgerald J.M., Sobieraj D.M., Holle L.M. (2021). Integration of a Virtual Dispensing Simulator “MyDispense” in an Experiential Education Program to Prepare Students for Community Introductory Pharmacy Practice Experience. Pharmacy.

[41] Nguyen K.T., Dao M.L., Nguyen K.N., Nguyen H.N., Nguyen H.T., Nguyen H.Q. (2023). Perception of Learners on the Effectiveness and Suitability of MyDispense: A Virtual Pharmacy Simulation and Its Integration in the Clinical Pharmacy Module in Viet Nam. BMC Med. Educ..

[42] Slater N., Mason T., Micallef R., Ramkhelawon M., May L. (2023). Enabling Access to Pharmacy Law Teaching during COVID-19: Student Perceptions of MyDispense and Assessment Outcomes. Pharmacy.

[43] Knobloch J., Cozart K., Halford Z., Hilaire M., Richter L.M., Arnoldi J. (2024). Students’ Perception of the Use of Artificial Intelligence (AI) in Pharmacy School. Curr. Pharm. Teach. Learn..

[44] Kattan L., Moideen S., Abdelrahman A., Khabbaz S., Ibrahim A., Mraiche F. (2026). Artificial Intelligence in Pharmacy Education: A Scoping Review of Current Integration & Global Perceptions. Curr. Pharm. Teach. Learn..

[45] Cole J., Ruble M., Astle K., Tabulov C., Singleton J., Sunjic K. (2024). Integration of Artificial Intelligence (AI) in Skills-Based Pharmacy Courses. Pharm. Educ..

[46] Ejaz H., McGrath H., Wong B.L., Guise A., Vercauteren T., Shapey J. (2022). Artificial Intelligence and Medical Education: A Global Mixed-Methods Study of Medical Students’ Perspectives. Digit. Health.

[47] Gharib A.M., Peterson G.M., Bindoff I.K., Salahudeen M.S. (2023). Potential Barriers to the Implementation of Computer-Based Simulation in Pharmacy Education: A Systematic Review. Pharmacy.

[48] Al-Diery T., Hejazi T., Al-Qahtani N., ElHajj M., Rachid O., Jaam M. (2024). Evaluating the Use of Virtual Simulation Training to Support Pharmacy Students’ Competency Development in Conducting Dispensing Tasks. Curr. Pharm. Teach. Learn..

