# Supplementary material for: Enhancing Self-Care Consultation Skills in Pharmacy Education: Benefits of Virtual Patients and Artificial Intelligence—A Scoping Review
Source: Pharmacy (Basel). 2026 May 11;14(3):71. doi: 10.3390/pharmacy14030071 (PMC13214858; doi:10.3390/pharmacy14030071)
Supplement: Supplementary file 1 [file pharmacy-14-00071-s001.zip › File S1. Full Electronic Search Strategy.pdf]

Full Electronic Search Strategy

Database: PubMed
Date of Search: January 31, 2026

1. Search Terms and String

The search was constructed using three primary concepts: Pharmacy Education, Self-Care/Medication, and Technology (VP/AI)

Concept	Search Terms
Concept 1: Pharmacy education	"pharmacy students" OR "pharmacy education"
Concept 2: Self-Care	"self-medication" OR "self-care"
Concept 3: Technology	"virtual patient" OR "virtual simulation" OR "artificial intelligence" OR "AI"

Full Consolidated Search String: ("self-medication" OR "self-care") AND ("virtual patient" OR "virtual simulation" OR "artificial intelligence" OR "AI") AND ("pharmacy students" OR "pharmacy education")

2. Search Limits and Filters

- **Language:** Limited to English.
- **Publication Type:** Peer-reviewed original research articles.
- **Publication Year:** No restrictions were applied regarding the publication year.
- **Exclusion Filters:** Systematic reviews, meta-analyses, narrative reviews, case reports, editorials, and conference abstracts were excluded.
